# Expanding the Spectrum of Charles Bonnet Syndrome: Severe Psychiatric Manifestations Associated With Total Vision Loss

**DOI:** 10.7759/cureus.84849

**Published:** 2025-05-26

**Authors:** Justin S Yun, Gabriela Prieto

**Affiliations:** 1 Ophthalmology, David Geffen School of Medicine, University of California, Los Angeles, Los Angeles, USA; 2 Psychiatry, Olive View-UCLA Medical Center, Los Angeles, USA

**Keywords:** atypical psychosis, charles bonnet syndrome, schizoaffective disorder, secondary psychotic features, sensory deprivation, total bilateral blindness, visual hallucinations

## Abstract

Charles Bonnet syndrome (CBS) is typically characterized by complex, non-threatening visual hallucinations in patients with visual impairment who maintain insight that their perceptions are unreal. While primarily considered an ophthalmological phenomenon, recent evidence suggests that CBS may overlap with psychiatric disorders presenting with complex visual hallucinations and impaired insight, complicating both diagnosis and management. These interactions challenge the view of CBS as a benign and isolated condition, especially when symptoms become more entrenched, resemble psychosis, or co-occur with pre-existing psychiatric disorders. We present a new, atypical case involving a patient with CBS and evolving psychotic symptoms at the Olive View Medical Center in California. Our case highlights a 40-year-old female patient with bilateral blindness and a history of CBS, schizoaffective disorder, and repeated Lanterman-Petris-Short conservatorships. At presentation, she exhibited aggressive behavior, possible delusions, responses to internal stimuli, and significant difficulties in self-care. Treatment included a regimen of olanzapine (25 mg daily), haloperidol (15 mg daily), gabapentin (1800 mg daily), hydroxyzine (150 mg daily), and divalproex sodium (2000 mg daily), resulting in some symptom amelioration but persistent psychotic features. The patient’s ongoing conservatorship demonstrates the severity and chronicity of her condition. This case suggests that even in patients with total vision loss, CBS has the potential to evolve beyond isolated visual hallucinations to include severe psychiatric sequelae, including psychotic symptoms. These findings call for greater clinical vigilance, timely ophthalmologic and psychiatric consultation, and interdisciplinary management. Further research is needed to elucidate the neurobiological mechanisms linking sensory deprivation to complex hallucinations and psychiatric disturbances. An improved understanding of these processes may guide more accurate diagnostics and inform targeted interventions, ultimately improving outcomes.

## Introduction

Charles Bonnet syndrome (CBS) is a condition in which individuals with significant visual impairment experience complex visual hallucinations, usually while maintaining insight into their unreal nature [1,2]. Simple hallucinations (e.g., flashes, geometric patterns) contrast with complex hallucinations (e.g., faces, animals, full scenes). CBS affects approximately 10.2% of ophthalmic patients overall, with higher rates of up to 24.6% in vision rehabilitation populations and 15.8% in patients with age-related macular degeneration (AMD) [3-5]. CBS is frequently underreported, largely due to patients’ fear of being misdiagnosed with a psychiatric disorder, as well as lack of awareness among clinicians [1,2]. Known risk factors for CBS include advanced age, duration and severity of visual deprivation, and underlying ocular pathologies such as AMD, glaucoma, and other retinal diseases, and additional risk factors identified in the literature include female sex, reduced contrast sensitivity, and social isolation [1,3-5]. Although it has historically been considered a benign phenomenon, emerging evidence indicates that CBS can sometimes present with severe psychiatric manifestations. These manifestations may include pronounced emotional distress, delusional thinking, and psychosis-like symptoms, particularly in cases of profound visual loss [6-8]. The underlying mechanisms are not fully understood, but recent research has reported potential involvement of cortical disinhibition and the consequences of sensory deprivation [9,10]. Distinguishing CBS from primary psychosis is critical, as management pathways and prognoses differ substantially. Management is individualized according to the needs of the patient, often involving treating the underlying diseases. For instance, patients with CBS caused by macular degeneration reported resolution of hallucinations with photodynamic therapy, and patients with cataracts had resolution of CBS with cataract extraction and intraocular lens implant [11,12]. Other than reassuring measures, treatments include selective serotonin reuptake inhibitors, valproate, or antipsychotics, or transcranial direct current stimulation, depending on the severity [13-16].

Comorbid psychiatric disorders can exacerbate these symptoms, complicating both diagnosis and management [6,7]. Misdiagnosis can lead to inappropriate interventions, emphasizing the critical role ophthalmologists play in recognizing atypical presentations of CBS and coordinating early psychiatric consultation. This report presents the case of a patient with bilateral blindness who exhibited severe psychiatric sequelae of CBS, highlighting the necessity of an interdisciplinary approach [2,11,17].

## Case presentation

We describe the case of a 40-year-old female patient with an extensive past medical history of substance abuse, seizure disorder, hepatitis C, cryptococcal meningitis treated with a ventriculoperitoneal shunt, total bilateral blindness secondary to elevated intracranial pressure and advanced glaucoma, who presented to the ED with visual and auditory hallucinations. This patient was completely blind without light perception for an extensive period before presentation, with no recorded period of partial vision (visual acuity: no light perception bilaterally). She also had a documented diagnosis of CBS from 2019 confirmed by neuro-ophthalmology, with consistent visual hallucinations described as bridges and colors to present. At the time of presentation, she was experiencing vivid and distressing visual hallucinations, pronounced fear, and aggressive behavior. As her condition progressed over the 24 hours in the ED, she exhibited persistent responses to internal stimuli and gradually lost the ability to discern hallucinations from reality, developing fixed delusions and paranoia unrelated to prior hallucinations. The patient was consequently admitted to Olive View Medical Center following this acute psychiatric crisis. Although lorazepam was administered to help control her agitation and anxiety, her fear and aggressive behavior persisted.

Her treatment regimen included olanzapine 25 mg/day, haloperidol 15 mg/day, gabapentin 1800 mg/day, hydroxyzine 150 mg/day, and divalproex sodium 2000 mg/day based on the patient’s chart, which alleviated the patient’s symptoms during her prior hospitalization. Despite this comprehensive pharmacological approach, the patient continued to exhibit significant psychotic features, including marked paranoia, delusions, and hallucinations. Her diagnosis was refined to schizoaffective disorder following the emergence of mood‐incongruent psychotic features, including auditory hallucinations and persecutory delusions. Over time, her clinical picture became more complex due to the schizoaffective disorder, although persecutory delusions became more intermittent, resulting in lengthened psychiatric hospitalization and ultimately Lanterman-Petris-Short conservatorship. Due to her severe impairment in self-care and persistent psychiatric symptoms, she remained under conservatorship to ensure her safety and proper treatment. Non-pharmacological interventions, such as vision rehabilitation programs, ophthalmology consult, or psychotherapy, were not feasible primarily because of the patient’s persistent, severely agitated, and psychotic state.

## Discussion

In its classic form, CBS involves visual hallucinations in individuals with significant vision loss, while they generally retain insight that these images are not real. In this case, the patient presented with a more severe form of CBS that was characterized by intense emotional distress, delusional thinking, and aggressive behavior. Profound bilateral blindness may increase the likelihood of such severe psychiatric complications, as the absence of all visual cues can exacerbate cortical disinhibition and amplify hallucinations.

When CBS coexists with other psychiatric disorders, such as schizoaffective disorder, it can be difficult to distinguish hallucinations that stem from sensory deprivation from those driven by a primary psychotic process [2,11]. This overlap can result in diagnostic ambiguity and complicate management. It is unknown if this patient had complex visual hallucinations preceding the onset of persecutory delusions at the time of presentation in the ED, involving both processes. However, at the time of initial assessment, the patient only reported visual hallucinations, making CBS the likely contributor to the patient’s symptoms. In cases where severe delusions and paranoia emerge, it is critical to consider the contribution of both CBS and the underlying psychiatric condition, rather than attributing all symptoms to one or the other.

The complexity of managing severe psychiatric presentations of CBS emphasizes the importance of collaboration between ophthalmologists and psychiatrists. Cognitive and psychiatric assessments are necessary to rule out delirium, dementia, or primary psychiatric disorders, as CBS requires preserved cognition and insight. While mild to moderate CBS often responds to reassurance, optimization of remaining vision, and psychosocial support, severe forms of CBS require robust psychiatric treatment. The patient’s regimen, which included multiple antipsychotics, mood stabilizers, and anxiolytics, illustrates the need for intensive pharmacological intervention. Nevertheless, her persistent psychotic symptoms highlight the inherent challenges in treating severe CBS when it is compounded by comorbid psychiatric illness. We classify this presentation as CBS complicated by psychotic features, as the onset of complex visual hallucinations likely precipitated the development of persecutory delusions and disordered thought. This differentiates it from simple exacerbation of a known psychiatric illness and the need for clinicians to recognize the potential for CBS to drive new psychiatric pathology.

Given the patient’s total blindness and lack of family or social support, non-pharmacological interventions such as orientation and mobility training, a form of rehabilitation for individuals who are blind or visually impaired to help them move safely and independently, or psychotherapies such as CBT for distressing hallucinations were not pursued at the time of her acute crisis, which is a limitation of this case report. However, these modalities may become essential components for her long-term management once her psychiatric symptoms are better controlled.

Future longitudinal research is necessary to clarify the pathophysiological mechanisms that link total visual deprivation to severe psychosis-like features in CBS. Neuroimaging studies could shed light on the specific cortical circuits involved in hallucination generation when visual input is absent. Additionally, randomized controlled trials comparing various pharmacological and non-pharmacological interventions could guide the development of standardized treatment protocols. Beyond clinical management, we advocate for proactive psychoeducation initiatives within low vision and older adult populations to raise awareness of CBS, reduce stigma, and encourage early self-reporting of visual hallucinations.

## Conclusions

This case highlights that CBS can present with severe psychiatric manifestations, particularly in the context of profound bilateral blindness and coexisting psychiatric disorders. Ophthalmologists and psychiatrists must maintain a high index of suspicion for atypical presentations of CBS and collaborate closely to provide comprehensive care. Early psychiatric or ophthalmologic consultation, along with targeted treatments and eventual sensory rehabilitation, is vital to ensuring appropriate diagnosis and management. By improving our understanding of the complex interplay between vision loss and psychiatric symptoms, we can optimize therapeutic strategies and outcomes for patients facing this challenging condition. Finally, we advocate for the development of formal clinical guidelines to standardize diagnosis, interdisciplinary consultation, and treatment of severe CBS presentations.
